# A Rare Case of Rapidly Progressive Extragonadal Mature Cystic Teratoma Presenting With Chest Pain

**DOI:** 10.7759/cureus.36631

**Published:** 2023-03-24

**Authors:** Ayman Mohamed, Lisa De Rose, Talal Bazzi, Mark Benjamin

**Affiliations:** 1 Internal Medicine, Ascension St. John Hospital, Detroit, USA

**Keywords:** chest pain, rupture teratoma, hemoptysis, teratoma, mediastium

## Abstract

Mature cystic teratomas represent the most prevalent subtype of germ cell tumors affecting the ovaries. Typically, these neoplasms are benign and characterized by a slow growth pattern. Nevertheless, malignant transformation of these tumors is a rare event that may occur. Despite their typically indolent behavior, some cases may exhibit rapid growth rates and give rise to a variety of complications, including rupture and consequent manifestation of a wide range of clinical signs and symptoms. This report details the case of a 49-year-old woman who presented to the hospital with a chief complaint of chest pain. The onset of her symptoms occurred several days prior to admission and was associated with fatigue without shortness of breath. Imaging studies, including computed tomography angiography and magnetic resonance imaging of the chest, revealed a mediastinal mass measuring 5.9 x 7.4 cm in a cross-sectional area that displayed features consistent with a mature cystic teratoma, including the presence of soft tissue, fat, fluid, and areas of calcification. Notably, a prior computed tomography scan of the chest, performed 20 months before her presentation, did not reveal any evidence of masses. The patient subsequently underwent successful robot-assisted resection of the mediastinal mass, with complete resolution of her symptoms. Histopathologic examination of the excised mass confirmed the absence of malignancy.

## Introduction

Mature cystic teratomas are the most commonly encountered form of germ cell ovarian tumors, usually exhibiting a benign and indolent clinical course, but malignancy can arise in around 1-3% of cases [1,2]. Mature cystic teratomas typically exhibit a gradual growth pattern, with an average growth rate of approximately 1.8 mm per year [3]. However, certain instances of rapid tumor expansion have been documented in the literature. Notably, the rupture of these teratomas can give rise to diverse clinical manifestations that may resemble those of other pathologies [4]. This case report documents the clinical presentation of a 49-year-old female patient who complained of chest pain, subsequently discovered to harbor a mediastinal mature cystic teratoma, displaying a noteworthy rapid growth rate, without any signs of primary ovarian involvement. Remarkably, imaging conducted 20 months prior to her admission revealed no evidence of mediastinal abnormalities.

## Case presentation

A 49-year-old female with a past medical history of coronavirus disease 2019 (COVID-19) infection presented to the hospital with complaints of chest pain. The onset of the symptoms was noted four days before presentation, and the intensity had gradually increased over time. The nature of the pain was described as stabbing, radiating down her right arm, and not exacerbated by movement. On further inquiry regarding the symptoms, the patient reported fatigue but denied experiencing any fevers, chills, shortness of breath, abdominal pain, nausea, or vomiting. In terms of the patient's surgical history, she had undergone a vaginal hysterectomy and bilateral laparoscopic salpingectomy six years prior to the present episode, due to chronic pelvic pain that failed uterine artery embolization and ligation in the past. During the surgical intervention, several leiomyomas were also discovered. Pathology of the cervix specimen revealed a high-grade squamous intraepithelial lesion.

**Figure 1:**
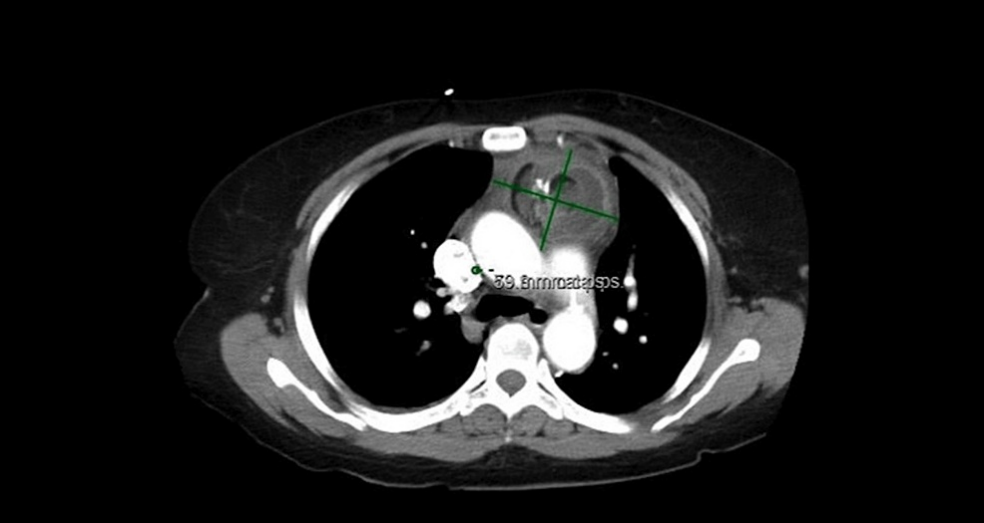
CT angiography of the chest revealing a mediastinal mass containing soft tissue, fat, fluid, and areas of calcification. CT: Computed tomography

Upon presentation, the patient's hemodynamic status was stable. Physical examination did not reveal any notable abnormalities. Table 1 shows the patient's lab values on admission. Computed tomography angiography of the chest and abdomen with IV contrast revealed a mediastinal mass measuring 5.9 x 7.4 cm in cross section, containing soft tissue, fat, fluid, and areas of calcification. The mass was situated along the anterior mediastinum, extending from the level of the manubrium down to the lower thorax (Figure 1). Computed tomography of the pelvis with contrast was performed to assess for a possible source of the mediastinal mass, but no masses were identified.

**Table 1:** Patient's lab values on admission.

WBC K/mcl	13
Sodium mmol/L	133
Troponin ng/mL	< 0.03
Alpha fetoprotein ng/ml	2.7
Lactate dehydrogenase IUnits/L	236
beta-HCG mIU/mL	< 1

**Figure 2:**
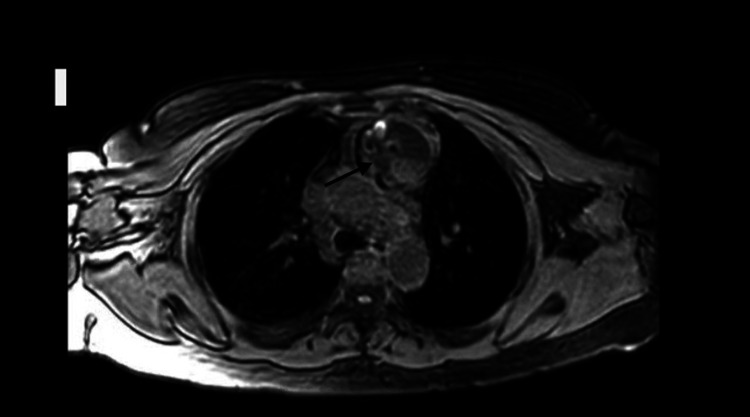
MRI of the chest with and without contrast confirming the presence of an anterior mediastinal mass measuring 5.9 x 7.4 x 8.5 cm. MRI: Magnetic resonance imaging

MRI of the chest with and without contrast confirmed the presence of an anterior mediastinal mass measuring 5.9 x 7.4 x 8.5 cm, which appeared to be cystic and solid, with fat components (Figure 2). Notably, the patient had undergone CT angiography of the chest at a different facility, approximately 20 months prior to presentation, for hypoxia secondary to COVID-19 pneumonia, which had not revealed any abnormalities or mediastinal masses.

Following diagnostic evaluation, the patient underwent robot-assisted resection to remove the mediastinal mass. The procedure was well-tolerated, and the patient experienced a resolution of her chest pain symptoms. Subsequent pathology examination of the excised mass showed cystic components consistent with a mature cystic teratoma containing a sebaceous soft material, mixed with hair and tooth. Notably, the excised mass was negative for malignancy.

## Discussion

The majority of germ cell tumors are known to develop within the gonads, whereas a smaller proportion of approximately 5-10% occur in other regions of the body, such as the mediastinum and retroperitoneum [5]. Teratomas, which are composed of at least two of the three mature germ cell layers, can be categorized into two groups. The first group comprises mature teratomas, which generally contain well-differentiated components and are typically considered benign; however, approximately 1-3% of cases may undergo malignant transformation. The second group encompasses immature teratomas, which consist of undifferentiated tissues [6]. In contrast, extra-gonadal germ cell tumors do not possess a discernible primary source in the ovaries or testes.

Patients with teratomas may present with a range of symptoms, including dyspnea, chest pain, fever, weight loss, and fatigue. Although mature teratomas typically exhibit slow growth rates, some cases of rapidly progressing teratomas have been reported. Notably, a prospective study examining the evolution of mature cystic teratomas found that the mean growth rate for teratomas was 1.8 mm per year [3]. Based on the imaging conducted less than two years prior, there were no observed mediastinal abnormalities for our patient. However, the accelerated growth rate detected is deemed atypical. The primary treatment for mature teratomas is radical resection [7]. However, in cases where complete resection is not feasible, subtotal resection may be performed to alleviate compressive symptoms.

Rupture is a rare but potentially serious complication of mature teratomas that necessitates prompt surgical intervention. Several reported cases have documented teratomas rupturing into adjacent structures, producing a variety of clinical presentations that may mimic lung malignancy or tuberculosis [4]. Teratomas may rupture into the tracheobronchial tree, resulting in hemoptysis and trichoptysis, or the lungs, leading to pneumonia and abscesses [8]. They may also rupture into the pleural space or the pericardium. Notably, a study comparing CT findings between ruptured and unruptured teratomas found that ruptured teratomas tend to exhibit a greater degree of inhomogeneity. Although the mean volume was larger in the ruptured group compared to the unruptured group, the results were not statistically significant [9]. The propensity of teratomas to rupture may stem from the secretion of proteolytic enzymes by the mass components or ischemia resulting from tumor growth, leading to necrosis and rupture.

## Conclusions

Mature cystic teratomas are known to present with various clinical manifestations that can resemble other pathological conditions, such as chest pain, shortness of breath, and hemoptysis, which can pose a challenge to clinicians. Therefore, it is imperative to consider the possibility of teratomas in the differential diagnosis, particularly in premenopausal females. The absence of detectable masses on previous imaging should not dissuade clinicians from considering teratomas as a potential diagnosis since some cases may exhibit an accelerated growth pattern, despite their typical slow growth tendency.
